# Assessment of genetic diversity and SNP marker development within peanut germplasm in Taiwan by RAD-seq

**DOI:** 10.1038/s41598-022-18737-0

**Published:** 2022-08-25

**Authors:** Yu-Ming Hsu, Sheng-Shan Wang, Yu-Chien Tseng, Shin-Ruei Lee, Hsiang Fang, Wei-Chia Hung, Hsin-I. Kuo, Hung-Yu Dai

**Affiliations:** 1 Université Paris-Saclay, CNRS, INRAE, Univ Evry, Institute of Plant Sciences Paris-Saclay (IPS2), 91405 Orsay, France; 2 Université Paris Cité, CNRS, INRAE, Institute of Plant Sciences Paris-Saclay (IPS2), 91405 Orsay, France; 3 Crop Science Division, Taiwan Agricultural Research Institute, Taichung, 413008 Taiwan, ROC; 4 Crop Improvement Division, Tainan District Agricultural Research and Extension Station, Tainan, 71246 Taiwan, ROC; 5 Agronomy Department, National Chiayi University, Chiayi, 60004 Taiwan, ROC

**Keywords:** Agricultural genetics, Genetic markers, Plant breeding, Next-generation sequencing

## Abstract

The cultivated peanut (*Arachis hypogaea* L.) is an important oil crop but has a narrow genetic diversity. Molecular markers can be used to probe the genetic diversity of various germplasm. In this study, the restriction site associated DNA (RAD) approach was utilized to sequence 31 accessions of Taiwanese peanut germplasm, leading to the identification of a total of 17,610 single nucleotide polymorphisms (SNPs). When we grouped these 31 accessions into two subsets according to origin, we found that the “global” subset (n = 17) was more genetically diverse than the “local” subset (n = 14). Concerning botanical varieties, the var. *fastigiata* subset had greater genetic diversity than the other two subsets of var. *vulgaris* and var. *hypogaea*, suggesting that novel genetic resources should be introduced into breeding programs to enhance genetic diversity. Principal component analysis (PCA) of genotyping data separated the 31 accessions into three clusters largely according to the botanical varieties, consistent with the PCA result for 282 accessions genotyped by 14 kompetitive allele-specific PCR (KASP) markers developed in this study. The SNP markers identified in this work not only revealed the genetic relationship and population structure of current germplasm in Taiwan, but also offer an efficient tool for breeding and further genetic applications.

## Introduction

Originated from South America, the cultivated peanut (*Arachis hypogaea* L.) is an allotetraploid (AABB, 2n = 4x = 40) and an important legume crop worldwide. Humans benefit from peanut seeds as food and source of oil due to their high percentage of proteins and fatty acids^1^. The annual production of peanuts has increased in the past 20 years to reach 53 million tons in 2020 according to FAOSTAT (http://www.fao.org/faostat). To fulfill the increasing peanut demand under the threat of climate change, breeding new varieties is an effective strategy to improve peanut qualitative and quantitative traits.

The conservation of *Arachis* germplasm and exploitation of their genetic diversity are crucial for the breeding of the cultivated peanut. Presently, several gene banks are renowned for their *Arachis* germplasm including the International Crops Research Institute for the Semi-Arid Tropics (ICRISAT), United States Department of Agriculture (USDA), and the Oil Crops Research Institute of the Chinese Academy of Agricultural Sciences (OCRI-CAAS). More than 15,000, 9,000 and 8,000 accessions were collected in ICRISAT, USDA, and OCRI-CAAS^2^, respectively. On the other hand, understanding the genetic diversity of in-hand germplasm is the prerequisite before launching breeding programs, and the utilization of molecular markers is the predominant strategy to evaluate the genetic diversity of germplasm at present^3^. Cultivated peanut has its low genetic diversity due to the recent hybridization of its two ancestors and selection in breeding programs^4–7^. Even though the narrow genetic diversity of cultivated peanuts has hindered the development of molecular markers, it has been possible to develop and utilize simple sequence repeat (SSR) markers to assess the genetic diversity in cultivated peanut^8–11^. In particular, the population structures of 92 accessions in the US Peanut Mini Core Collection and 196 major peanut cultivars in China were revealed by SSR markers^12,13^. Although SSR markers were widely used for identifying genetic diversity of peanut populations, these studies had limited population size due to the challenging genotyping process.

Recently, the peanut genome projects made possible by next generation sequencing (NGS) have revolutionized genetic research in cultivated peanuts. So far, the genomes of *Arachis hypogaea* L. and its two diploid ancestors, *A. duranensis* (AA) and *A. ipaensis* (BB), have been sequenced^6,7,14^. These high quality genome sequences have paved the way for developing high-throughput single nucleotide polymorphism (SNP) markers e.g. via genotyping-by-sequencing (GBS) that can then facilitate peanut molecular breeding. The 58 K SNP array ‘*Axiom_Arachis*’, developed by resequencing 41 peanut accessions, was used to identify genetic diversity across 384 *Arachis* genotypes including USDA Mini Core Collection and wild species^15,16^, while 787 accessions from the U.S. Peanut core collection were genotyped by the 14 K ‘*Arachis_Axiom2*’ SNP array to reveal their genetic diversity^17^. Compared to SNP arrays, GBS is a more cost-effective technique based on sequencing of the reduced genome associated with restriction sites using NGS^18,19^. In peanut research, this technique was applied in SNP development, enabling the construction of genetic maps for quantitative trait locus (QTL) mapping and the analysis of population structure^20–22^.

In Taiwan, peanut breeding programs can be traced back to the late 1950s. To date, most varieties developed locally have been obtained by conventional breeding based on evaluating morphological traits. In such breeding programs, the parental selection mainly relied on the pedigree information or the collection source to infer genetic relationships. Thus, exploiting available molecular tools to characterize the present peanut varieties in Taiwan should allow improved breeding programs in the future. In this study, we performed the restriction site-associated DNA (RAD) approach to sequence 31 genotypes—including current elite varieties developed in Taiwan and important accessions introduced from abroad—to reveal the underlying genetic diversity. Furthermore, 14 kompetitive allele-specific PCR (KASP) markers were designed and then used to genotype 282 other accessions. Overall, this work reveals the genetic structure of peanut germplasm in Taiwan through SNP markers identified by RAD-seq and these markers can be used for a number of applications such as variety identification and breeding programs.

## Materials and methods

### Plant materials and DNA extraction

31 peanut accessions, maintained by Taiwan Agricultural Research Institute (TARI) and Tainan District Agricultural Research and Extension Station (Tainan DARES), were chosen for RAD-seq construction. These accessions consist of elite cultivars, advanced breeding lines, and “introduced” old accessions acquired in South American countries close to the geographic origin of peanut (Supplementary Table S1). Among 31 peanut accessions, there are 13 Spanish, 11 Valencia, 3 Virginia, and 4 Runner type accessions. For the genotyping via KASP markers, 282 peanut accessions were obtained from the National Plant Genetic Resources Center in TARI, including 66 Spanish, 27 Valencia, 49 Virginia and 88 Runner type accessions. The plant materials utilized in this study conform to relevant international, national and institutional guidelines.

The DNA extraction of all accessions was based on young leaves collected from seedlings within two weeks using the modified CTAB method which replaces phenol and chloroform with potassium acetate to remove protein and polysaccharides^23^. The DNA samples extracted from the modified CTAB method can directly be used for KASP genotyping, but need further purification to ensure their quality for RAD-seq library construction. Thus, after extracting DNA of 31 accessions used for RAD-seq, we utilized the QIAGEN kit (DNeasy Blood & Tissue Kit; Qiagen, https://www.qiagen.com/, Hilden, Germany) to purify these DNA samples which were then quantified and qualified by NanoDrop 2000 spectrophotometer (Thermo Fisher Scientific Inc., https://www.thermofisher.com, DE, USA). The purified DNA samples with (1) the 260/280 ratio from 1.8 to 2.0, (2) the 260/230 ratio from 2.0 to 2.4, and (3) the concentration ≥ 25 ng/µl were further checked for the DNA integrity by agarose gel (1.0%) electrophoresis.

### Phenotypic evaluation

24 out of the 31 peanut accessions used in RAD-seq and 282 additional peanut accessions of the germplasm were phenotyped in the fall of 2016 in TARI (coordinates 24° 01′ 47.5″ N 120° 41′ 47.4″ E), and 20 plants of each accession were evaluated for 8 quantitative traits, including days to flowering (between the sowing and flowering date), plant architecture, number of pods, yield (g/m^2^), 100-pod weight, 100-seed weight, rust resistance and leaf spot resistance. The susceptibility to these two peanut diseases was quantified under natural conditions in the field since these diseases develop spontaneously during the fall, and the disease symptoms were scored from 1 (having no symptoms) to 9 (highly susceptible)^24^. Depending on the degree of inclination from verticality, plant architecture was scored from 0 (the most upright) to 9 (the most prostrate).

### RAD-seq library construction and SNP calling

After finalizing DNA extraction, purification and quality control of 31 peanut accessions, we used 1.1 µg of each high-quality DNA sample to make two RAD-seq libraries from 16 and 15 accessions, respectively, following the published protocol, and *PstI* was chosen as the digestion enzyme^25^. Next-generation sequencing of each library was carried out in the Genome Research Center of Yang-Ming University using the Illumina Hiseq 2500 platform (Illumina Inc., https://www.illumina.com, CA, USA) with 100 bp single-end reads in two lanes. The sequencing data have been deposited at National Center for Biotechnology Information (NCBI) under BioProject PRJNA811600. For SNP calling, single-end reads were first debarcoded by Stacks using the program “process_radtags”^26^. Then, we used Burrows-Wheeler Alignment (BWA) v0.7.17-r1188 “aln” to align the reads of each accession onto the reference genome of cultivated peanut and its diploid ancestors for identifying SNPs used in the genetic diversity analysis and the development of KASP markers, respectively^6,14,27^. When the genome of cultivated peanut was published^14^, all of the KASP markers used in this study had already been designed using the merged genomes of two diploid ancestors of cultivated peanut^6^. Thus, identified SNPs based on the genome of cultivated peanut were only used for the *in-silico* analyses that investigated the genetic diversity of the 31 accessions, but in all cases the SNP calling pipeline was the same. After the alignment was finished, Samtools and BCFtools were utilized for SNP calling and filtering, SNPs were kept with (1) base quality ≥ 20, (2) mapping quality score ≥ 20, and (3) depth ≥ 3. Then, a customized R script was used to create Variant Call Format (VCF) files encompassing qualified SNPs that discriminated the 31 accessions^28^.

### The development and validation of KASP markers

Among SNPs available for distinguishing 31 peanut accessions based on the genome of the two diploid ancestors^6^, we extracted 1,230 homozygous SNPs with informative alleles in all 31 accessions, and then discarded 783 SNPs having Polymorphic Information Content (PIC) values lower than the average over all SNPs. Finally, 29 out of 477 SNPs with an average PIC value of 0.28 were selected for developing KASP markers. These 29 putative SNPs with 100 bp flanking sequences on both sides were used for designing KASP primers that were then synthesized by LGC genomics (http://www.lgcgroup.com, Teddington, England). The validation of KASP markers was performed on the 96-well StepOnePlus™ Real-Time PCR System (Thermo Fisher Scientific Inc., https://www.thermofisher.com, DE, USA), and each 10-μL reaction consisted of 12.5 ng of DNA, 0.14 μL of KASP assay mix and 5 μL of KASP Master Mix (2X). The PCR protocol was carried out as follows: (1) pre-read stage at 30 °C for 1 min, (2) hold stage at 94 °C for 15 min, (3) PCR stage 1 of 10 touchdown cycles using 94 °C for 20 s and 61 °C (decreasing 0.6 °C per cycle) for 1 min, (4) PCR stage 2 with 26 amplification cycles at 94 °C for 20 s and 55 °C for 1 min, and (5) post-read stage at 30 °C for 1 min. When the PCRs were completed, the fluorescent signals of samples were analyzed by the StepOne™ software for determining genotypes.

### Statistical analysis

All statistical analyses were carried out in R 3.63. The PIC value and the expected heterozygosity (He) were determined for each SNP marker^29,30^. Principal component analysis (PCA) using phenotypic data was performed by the “PCA” function in the “FactoMineR” package^31^. In the “poppr” package, the “bitwise.dist” function was used to calculate the genetic distances between the 31 accessions, and these distances were calculated depending on the fraction of loci which differ between germplasm^32,33^. The “aboot” function was utilized to construct the dendrograms based on the unweighted pair group method with arithmetic mean (UPGMA) with 1000 bootstraps. In the “adegenet” package, PCA for SNP data from 31 accessions was carried out by the “glPCA” function, and population structure of 282 accessions was addressed by successive K-mean clustering and discriminant analysis of principal components (DAPC) using the “find.clusters” and “dapc” function, respectively^34,35^. In addition to the dendrogram plot, created by the “plot.phylo" function in the “ape” package^36^, all visualization was performed using the “ggplot” function in the “tidyverse” package^37^.

## Results

### SNP marker development from 31 peanut accessions using RAD-seq

In this study, 31 peanut accessions were chosen to conduct RAD-seq, of which 17 accessions were introduced from abroad and 14 accessions developed or collected in Taiwan. This collection has important agronomic traits including yield-related traits, resistances to biotic and abiotic stresses, and valuable characteristics at the genetic diversity level (Supplementary Table S1).

In the RAD-seq approach, the six-cutter enzyme, *PstI*, was utilized for the DNA digestion, and so sequencing of each accession focused on approximately 5% (100-bp extensions on both side of a *PstI* cutting site that occurs every 4,096 bp on average) of the total cultivated peanut genome (2.7 Gb). The estimated sequencing depth in the 31 accessions ranged from 4.26 (HL2) to 15.01 (Red), and the average depth was 9.47. In addition, more than 99.0% of sequenced reads from all samples were properly aligned to the reference genome. Compared to the reference genome, *A. hypogaea* cv. Tifrunner, there were 1475 to 14,471 SNPs identified from these 31 accessions with an average of 5249 SNPs, and more than 3 quarters of these polymorphisms were homozygous. In addition, the transition/transversion (Ts/Tv) ratio ranged from 0.48 to 1.19 (Table 1). In terms of the three botanical varieties of the cultivated peanut, accessions from subsp. *fastigiata* var. *vulgaris* (Spanish type)*,* subsp. *fastigiata* var. *fastigiata* (Valencia type) and subsp. *hypogaea* var. *hypogaea* (Virginia/Runner types) had a total of 5006, 5119 and 5905 SNPs, i.e., the differences across botanical varieties were very small. Interestingly, the 31 accessions separated well according to the global and local collection, corresponding to 17 genotypes introduced from other countries and 14 genotypes from Taiwan, respectively. The global collection had an average of 6071 SNPs which was higher than the average of 4526 SNPs for the local collection. Moreover, 8 introduced accessions, collected in South America close to the center of origin of cultivated peanut, led to an average of 7139 SNPs, even higher than that of the global collection. This result suggested that the global collection germplasm from various countries had more polymorphisms than the local one containing mainly Taiwanese cultivars.

**Table 1:** Sequence and SNP information of our 31 accessions in the Taiwanese peanut germplasm.

Germplasm	Properly mapped reads (%)	Estimated depth^a^	Filtered SNPs^b^	Homozygous SNPs^b^	Ts/Tv ratio^c^
PI153169	99.44	8.4	5666	5342	0.66
PI259717	99.26	8.98	5502	5118	0.67
PI565455	98.99	10.65	14,471	14,051	0.63
Tainung 7 (TNG7)	99.52	12.04	6166	5848	0.60
Tainung 10 (TNG10)	99.56	12.14	5884	5630	0.64
Tainan 14 (TN14)	99.18	9.7	1682	1348	1.12
Tainan 15 (TN15)	99.11	6.38	1475	1217	1.19
Tainan 18 (TN18)	99.15	5.02	1794	1461	0.68
Tainan Selection 9 (TNS 9)	99.04	13.13	6884	6387	0.59
Hualieng 1 (HL1)	99.14	9.77	3600	3230	0.67
India	99.09	10.67	3495	3174	0.72
Xiamen	99.02	11.35	5858	5483	0.70
Vietnam	99.10	7.97	2605	2301	0.75
PI118480	99.48	9.25	5564	5277	0.83
PI118989	99.28	11.02	11,823	11,367	0.53
PI155112	99.44	7.87	5163	4829	0.74
PI314817	99.38	9.16	11,051	10,653	0.73
PI338337	99.62	10.72	6187	5830	0.69
Tainan 16 (TN16)	99.15	7.63	1683	1326	1.19
Tainan 17 (TN17)	99.19	4.77	1606	1324	1.14
Hualieng 2 (HL2)	99.17	4.26	1896	1642	0.62
E01001	99.22	6.32	2376	1923	0.98
E01004	99.21	6.05	2759	2393	0.85
Red	99.17	15.01	6204	5797	0.51
NS011001	99.15	9.97	4147	3735	0.87
PI109839	99.57	10.19	6805	6499	0.48
Taichung 1 (TC1)	99.63	12.65	7390	7097	0.65
PI145681	99.47	9.51	2618	2342	0.56
PI599592	99.55	12.68	6408	6150	0.69
PI203396	99.59	8.92	4855	4593	0.59
Penghu 1 (PH1)	99.34	11.37	9115	8713	0.58

^a^The estimated depth was calculated by the total number of bases divided by 4.8% of 2.7 Gb, the size of reduced reference genome.

^b^The SNP identification was based on the reference genome of *A. hypogaea* cv. Tifrunner.

^c^Ts/Tv is the abbreviation of transition/transversion.

Then, the next stage of filtration was performed to keep only SNPs differentiating these 31 accessions. As a result, 3474 out of 17,610 SNPs were finally kept for the genetic diversity analysis using a tolerance of 6 missing values (20%) at most for each polymorphism.

### Evaluation of genetic diversity and cluster analysis based on 31 peanut accessions

**Figure 1:**
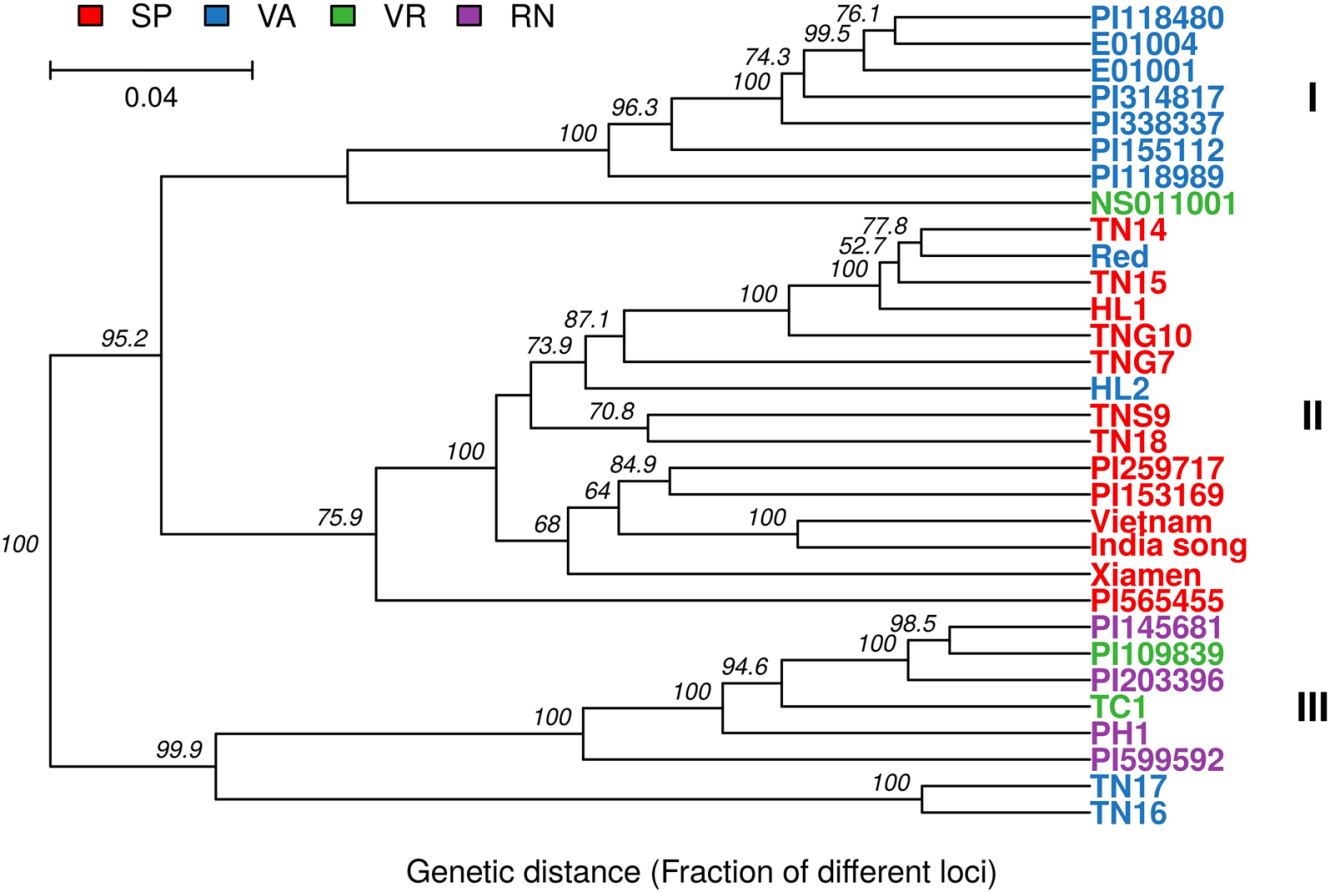
Dendrogram of the 31 accessions created from the unweighted pair group method with arithmetic mean (UPGMA). This dendrogram was based on the pairwise genetic distance with 1000 replicates using 3474 single nucleotide polymorphisms (SNPs). The branch length represents genetic distance, and the scale is on the top left of this figure. The numbers on the branches are bootstrap percentages. The legend shows the color of four market types, Spanish (SP), Valencia (VA), Virginia (VR), Runner (RN), and three clades clustered in this plot were named as I, II and III.

The genetic diversity of the 31 peanut accessions was quantified by a number of measures, including the expected heterozygosity (He), the major allele frequency (MAF), polymorphic information content (PIC), and genetic distance. The genetic distance was based on the bitwise distance, identical to Provesti's distance, growing with the fraction of genetically different loci between 31 accessions^32^. The pairwise comparison of the genetic distance between accessions is listed in Supplementary Table S2. On average, these 31 peanut accessions had a He of 0.19, PIC of 0.16, MAF of 0.87, and distance of 0.17. While considering botanical varieties, germplasm from subsp. *fastigiata* var. *fastigiata* had the largest average He, PIC and genetic distance (He = 0.18, PIC = 0.15, distance = 0.15) and smallest MAF (0.87), to be compared to that of the germplasm from subsp. *fastigiata* var. *vulgaris* (He = 0.13, PIC = 0.11, MAF = 0.90, distance = 0.11) or subsp. *hypogaea* var. *hypogaea* (He = 0.12, PIC = 0.10, MAF = 0.92, distance = 0.11) (Table 2), showing that Valencia type germplasm acquired higher genetic diversity than both Spanish type and Virginia/Runner type germplasm. In terms of the collection source, the global collection had larger average He, PIC and genetic distance (He = 0.19, PIC = 0.15, distance = 0.17) and smaller MAF (0.86) than the local collection (He = 0.16, PIC = 0.14, MAF = 0.88, distance = 0.14), indicating that the global collection had greater genetic diversity than the local collection. In addition, the distance tree for cluster analysis was reconstructed based on the UPGMA method with 1,000 bootstraps (Fig. 1). The results showed that 26 out of the 31 accessions were clustered into 3 groups mainly according to three botanical varieties. Germplasm of subsp. *fastigiata* var. *fastigiata* and subsp. *fastigiata* var. *vulgaris* were clustered into Group I and II with a distance of 0.19, and germplasm of subsp. *hypogaea* var. *hypogaea* was clustered into Group III separated from Group I and II with a distance of 0.21. With the exception of 5 accessions, NS011001 (Virginia type) was clustered into group I with mainly Valencia type germplasm, while two Valencia type accessions, Red and HL2, were clustered into group II with mostly Spanish type germplasm. Interestingly, TN16 and TN17, two Valencia type cultivars, were clustered into group III, but they were separated from accessions of subsp. *hypogaea* var. *hypogaea* with a distance of 0.18.

**Table 2:** Genetic diversity in the 31 accessions of Taiwanese peanut germplasm.

	Number	Mean He	Mean MAF^a^	Mean PIC^b^	Mean genetic distance
The whole collection	31	0.19	0.87	0.16	0.17
**The origin of germplasm**					
The global subset	17	0.19	0.86	0.15	0.17
The local subset	14	0.16	0.88	0.14	0.14
**The botanical variety**					
var. *vulgaris*	13	0.13	0.90	0.11	0.11
var. *fastigiata*	11	0.18	0.87	0.15	0.15
var. *hypogaea*	7	0.12	0.92	0.10	0.11

^a^MAF, major allele frequency.

^b^PIC, polymorphic information content.

**Figure 2:**
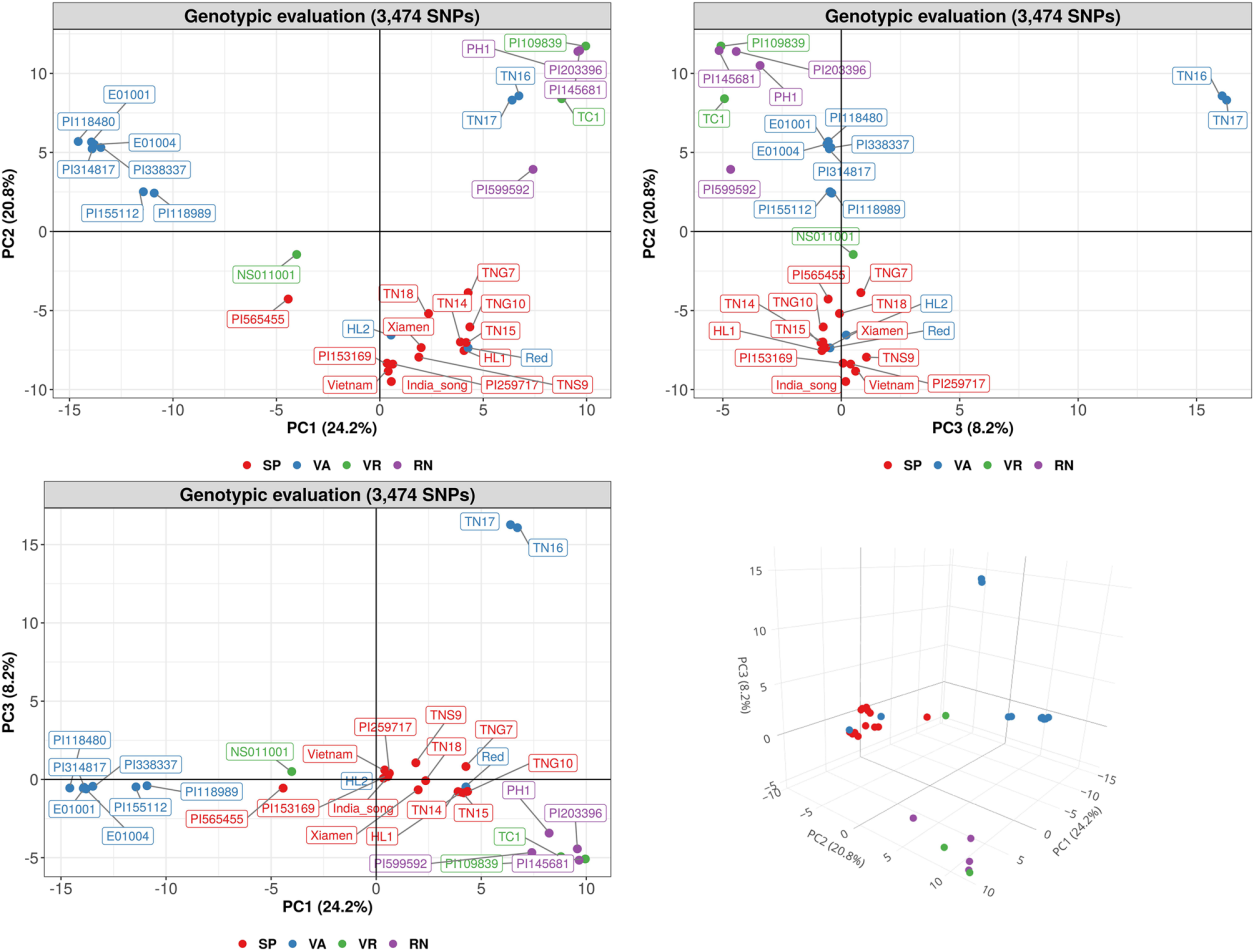
Principal component analysis (PCA) of the 31 accessions based on 3474 single nucleotide polymorphisms (SNPs). The 31 accessions were visualized by 4 market types, Spanish (SP), Valencia (VA), Virginia (VR), Runner (RN).

To further investigate and compare the genetic relationship among these germplasm, PCA were performed using genetic distances between the 31 accessions calculated via 3474 SNPs. The PCA result showed that the first three principal components (PC1, PC2 and PC3) explained 24.2%, 20.8% and 8.2% of the variance, respectively, totaling 53.2% of the overall genetic distance variance (Fig. 2). However, the scatter plots of PCs suggested that PCA based on genomic data distinguished well the 31 accessions. In the three biplots of PC1/PC2, PC2/PC3 and PC1/PC3 based on PCA using 3474 SNPs, the first pair succeeded in distinguishing 31 accessions into three clear groups, mainly according to three botanical varieties, subsp. *fastigiata* var. *vulgaris* (Spanish type)*,* subsp. *fastigiata* var. *fastigiata* (Valencia type) and subsp. *hypogaea* var. *hypogaea* (Virginia/Runner types), while the second and third pair were capable of separating TN16 and TN17 from three clusters assigned by the first pair (Fig. 2). Furthermore, the 3D scatter plot created using the three PCs displayed a relationship of 31 accessions compatible with the three PC biplots (Fig. 2).

### The development and validation of KASP markers

In this study, one of our goals was to design a set of non-gel based SNP markers which could be exploited to investigate the genetic structure within the germplasm collection conserved in the National Plant Genetic Resources Center of TARI. When this project was launched, the genome of cultivated peanut was not published yet. Thus, the development of SNP markers for the KASP genotyping relied on the two diploid ancestors of cultivated peanut^6^. Note that the SNP calling pipeline used here was the same as the one that identified SNPs from the cultivated peanut genome for assessing the genetic diversity of the 31 accessions. Of the SNPs identified by the mapping to the two diploid ancestral genomes, 1230 had both alleles represented in the 31 accessions while satisfying the constraint of being homozygous and having no missing data therein. 477 of these SNPs were kept because their PIC value was higher than the average one of the 1230 homozygous SNPs (Supplementary Table S3). At the same time, we conducted a field experiment in the fall of 2016 to evaluate 8 agronomically quantitative traits for 24 of the 31 accessions used in RAD-seq and the other 282 peanut accessions of the TARI germplasm with 66 Spanish, 27 Valencia, 49 Virginia and 88 Runner type accessions. The summary statistics indicated that accessions from subsp. *fastigiata* had early maturity characteristics, more pods and higher yield but slightly less spot resistance compared to accessions from subsp. *hypogaea* (Supplementary Table S4). The phenotypic data from this trial enabled us to compare the capability of genotypic and phenotypic data to identify genetic relationships between peanut accessions (Supplementary Table S5).

**Figure 3:**
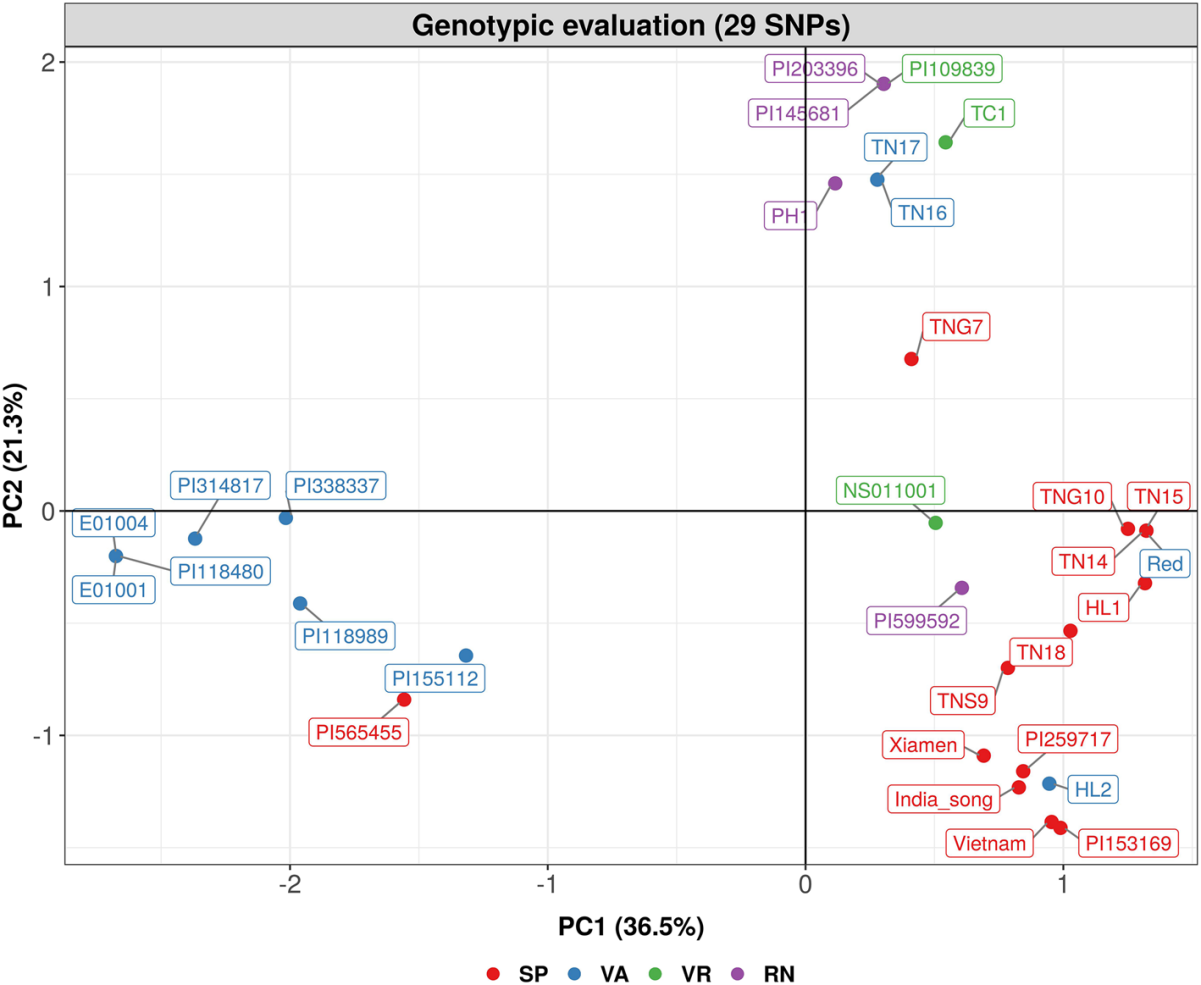
Principal component analysis (PCA) of the 31 accessions based on 29 single nucleotide polymorphisms (SNPs). 31 accessions were visualized by 4 market types, Spanish (SP), Valencia (VA), Virginia (VR), Runner (RN).

We performed PCA separately for the genotypic data from 29 out of 477 SNPs with an average PIC value of 0.28 and for the phenotypic data (8 agronomic traits from 24 of the 31 accessions based on field experiments). For PCA based on 29 SNPs, 31 accessions were grouped into three clusters according to their botanical varieties (Fig. 3). In addition, with eigenvalues between 0.49 and 1.79, the first three PCs accounted for 67.8% of total variance (Supplementary Table S6). The top three SNP markers having the most contribution to three PCs were as follows: (1) PC1: B02_105774702, B04_1643180, B09_70140267 and B09_141920571, (2) PC2: A01_9265671, A02_65802170 and A01_90269752 and (3) PC3: B05_133797191, A09_45155599 and A01_90916564 (Supplementary Table S6). On the other hand, PC1, PC2 and PC3 in the PCA that relied on the phenotypic data of 8 agronomic traits cumulated 73.0% of the overall phenotypic variance, and these PCs had eigenvalues ranging from 1.46 to 2.41 (Supplementary Table S7). The top three traits contributing to three PCs the most were as follows: (1) PC1: yield, number of pods and days to flower, (2) PC2: plant architecture, number of pods and 100 seed weight and (3) PC3: leaf spot level, rust level and 100-pod weight (Supplementary Table S7). Unlike the PCA result using 29 SNPs, for the three biplots and 3D scatter plot from the PCA depending on phenotypic data, none provided much evidence for structure within the 24 of the 31 accessions (Supplementary Fig. S1), suggesting that 29 SNPs were able to better distinguish 31 accessions than 8 agronomic traits. These 29 SNPs were therefore designed as KASP markers.

**Figure 4:**
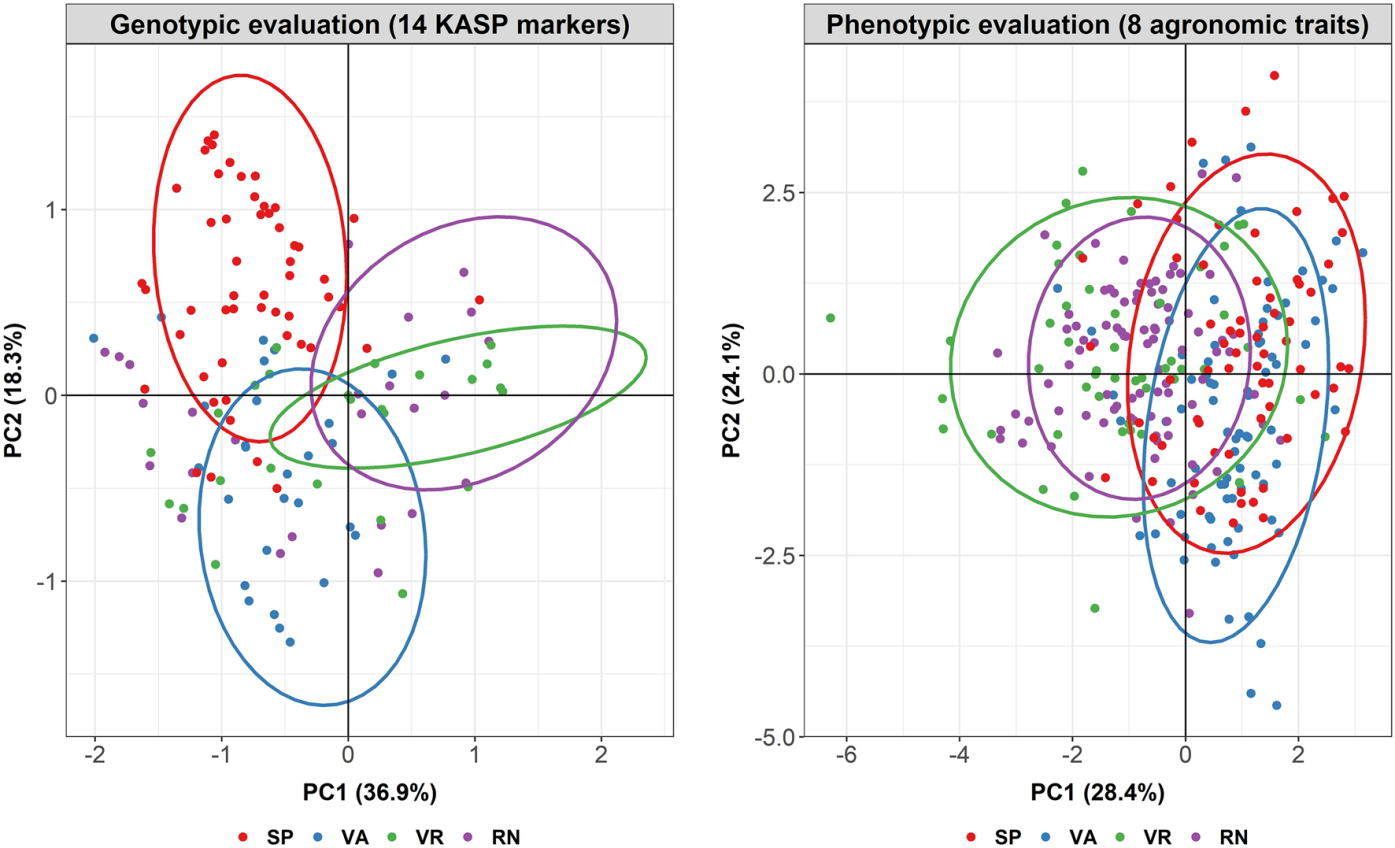
Principal component analysis (PCA) of 282 accessions based on genotyping data of 14 kompetitive allele-specific PCR (KASP) and phenotypic data. 282 accessions were visualized by 4 market types, Spanish (SP), Valencia (VA), Virginia (VR), Runner (RN).

To validate these 29 KASP markers, 282 accessions of the TARI germplasm with 66 Spanish, 27 Valencia, 49 Virginia and 88 Runner type accessions were genotyped. 14 out of 29 KASP markers showed a stable and discernible genotyping result in the initial validation process. The population structure analysis of 282 accessions was then determined by PCA using either genetic distances between these 282 accessions calculated by the KASP-marker genotyping data (Supplementary Table S8) or the phenotypic data from the field experiment in the fall of 2016 based on 8 agronomic traits. The PCA biplots indicated that the PCA using the genotypic data performed better than the one using the phenotypic data to distinguish 282 accessions. For the scatter plots based on genomic data, PC1 and PC2 explained 36.9% and 18.3% of the variance of the genotyping data and separated these accessions into 3 groups according to three botanical varieties (subsp. *fastigiata* var. *vulgaris,* subsp. *fastigiata* var. *fastigiata* and subsp. *hypogaea* var. *hypogaea*). In addition, the KASP markers mostly contributing to the variance of PC1 and PC2 were B04_84804214, B09_6670331, A01_9265671, A02_65802170 and A05_80673567, A01_90916564 (Supplementary Table S9). On the other hand, the first two PCs from the PCA using phenotypic data accounted for 28.4% and 24.1% of phenotypic variation, and only quite roughly separated these accessions into two groups (subsp. *fastigiata* var. *vulgaris*/var. *fastigiata* and subsp. *hypogaea* var. *hypogaea*). Most Spanish and Valencia type accessions were difficult to distinguish using phenotypic data, and the grouping between Spanish/Valencia and Virginia/Runner accessions was less clear than in the PCA result based on genotyping data (Fig. 4). The major traits accounting for the variance of PC1 and PC2 were days to flowering, leaf spot level, yield and number of pods (Supplementary Table S10).

To further identify the population structure within the 282 accessions, discriminant analysis of principal components (DAPC) was performed based on increasing number of clusters (K) assigned by successive K-means. In such an approach, the Bayesian information criterion (BIC) was used to assess the model relevance, and the result showed that it was best to go to values of K of at least 3 for clustering the 282 accessions (Supplementary Fig. S2). Similarly, DAPC was conducted for 2, 3 and 4 clusters to explore the population structure of 282 accessions. At K = 2, clusters corresponded to Spanish/Valencia and Virginia/Runner type accessions. At K = 3, the 3 groups corresponded largely to Spanish, Valencia and Virginia/Runner. At K = 4, the overall grouping trend was similar to that with K = 3, but had a mixture of accessions from the three botanical varieties that were assigned into the fourth group (Supplementary Fig. S3). This result suggested that our KASP markers are effective for identifying the population structure of peanut germplasm according to the botanical varieties, and it can even illustrate the similar genetic background acquired by accessions corresponding to a mixture of botanical varieties.

## Discussion

### The worldwide peanut accessions accumulate more polymorphisms

Molecular markers are of importance in many aspects of plant genetics and breeding, including variety identification, positional cloning, and the exploration of genetic diversity and population structure within germplasm. Developing molecular markers in the cultivated peanut was challenging because of its narrow genetic diversity and the high sequence similarity between its two diploid genomes^38^. The assembled genomes of cultivated peanuts and their diploid ancestors using NGS approaches has significantly boosted the genomic research in the peanut community. In particular, reduced-representation sequencing, such as GBS and RAD-seq, has been widely used in peanut research^20–22^. In this study, the RAD-seq approach was utilized to sequence 31 accessions of Taiwanese germplasm, decomposed into a “global” subset containing 17 “introduced” accessions and a “local” subset containing 14 Taiwanese accessions, 12 being current elite cultivars, 1 being a landrace, and 1 being an advanced breeding line. The global subset had a higher average number of SNPs than the local subset, suggesting that the germplasm from abroad had more polymorphisms than the local germplasm, and this can be explained by the fact that the accessions of the global subset were mainly introduced from North and South America encompassing the region of origin of domesticated cultivated peanut, supporting the idea that the origin of domesticated crops accumulates high diversity^39^. This result was compatible with previous work in soybean and sorghum based on SSR markers. Indeed, Iquira et al.^40^ and Ghebru et al.^41^ both found that the germplasm collection with accessions mostly from the origin of their cultivated crop had more unique alleles than the other collection with accessions from regions distant from the origin.

### The introduced accessions are more genetically diverse than the local ones

The genetic diversity of these 31 accessions was then investigated using several approaches based on 3474 SNPs. As a whole, this panel had an average PIC, expected He, MAF and genetic distance of 0.16, 0.19, 0.87, 0.17 (Table 2), respectively, which was concordant with previous research using SNP genotyping^42–44^. Note that the PIC value is a marker’s level of polymorphism. Markers are considered as highly informative (greater than 0.5), reasonably informative (0.25–0.5) and only slightly informative (smaller than 0.25) according to their PIC values^29^. The average PIC of 0.16 from these 31 accessions fell in this last class, while 32% of the identified SNPs corresponded to the reasonably informative class. In studies of three other germplasm collections genotyped by 48 K and 58 K SNP arrays, two collections comprising accessions of three botanical varieties like this study had a mean PIC value of 0.19^42,44^, and the third germplasm collection, having only accessions from two botanical varieties, had the mean PIC value of 0.08^43^, implying that the germplasm panel of 31 accessions chosen in this study preserves a high proportion of overall genetic diversity in spite of a smaller sample size compared to the ones in these three studies.

Focusing on subsets associated with the origin of our germplasm, Table 2 showed that the global subset (n = 17) had a higher average PIC value (0.15) than the local subset (0.14 with n = 14). Similarly, the average He and genetic distance of the global subset (He = 0.19, distance = 0.17) were greater than that of local subset (He = 0.16, distance = 0.14), indicating that the global subset had larger genetic diversity than the local subset. While these 31 accessions were separated into three botanical varieties, accessions from subsp. *fastigiata* var. *fastigiata* (Valencia type) had larger He (0.18), PIC (0.15) and genetic distance (0.15) on average than subsp. *fastigiata* var. *vulgaris* (Spanish type, He = 0.13, PIC = 0.11, distance = 0.11) and subsp. *hypogaea* var. *hypogaea* (Virginia/Runner types, He = 0.12, PIC = 0.10, distance = 0.11). In the 31 accessions, 11 genotypes were from subsp. *fastigiata* var. *fastigiata* including 7 introduced accessions, 3 cultivars and 1 landrace; in particular, 5 of 7 introduced accessions were from countries in South America including Brazil, Uruguay, Peru and Venezuela. The cultivated peanut originated from South America^45^, it is thus expected that genotypes of subsp. *fastigiata* var. *fastigiata* have larger genetic diversity than genotypes of subsp. *fastigiata* var. *vulgaris* and subsp. *hypogaea* var. *hypogaea* composing most cultivars in Taiwan. They also are expected to have higher diversity than this study’s introduced accessions coming from regions away from the center of origin of cultivated peanut.

### The peanut varieties developed in Taiwan may suffer from genetic vulnerability

Genetic relationships among the 31 accessions were investigated using the pairwise genetic distance for the construction of the dendrogram and PCA (Supplementary Table S2). In general, these 31 accessions were grouped into three clusters in line with three botanical varieties. Group I and Group II with mainly subsp. *fastigiata* var. *fastigiata* and subsp. *fastigiata* var. *vulgaris*, respectively, were separated by a genetic distance of 0.19, and Group III was separated from Group I/II by a genetic distance of 0.21 (Fig. 1). These results indicated that Group I and Group II were more closely related to one-another than to Group III, in agreement with the botanical classification and with other studies having larger sample sizes^12,16,22^.

This dendrogram result also suggested that the local cultivars in Taiwan might be suffering from low genetic diversity due to the excessive exploitation of narrow genetic resources as breeding material, notably genotypes of Spanish type germplasm (Fig. 1). In the main clade within Group II, there were 9 accessions from the local subset containing 8 cultivars and 1 landrace. According to the pedigree of these 8 cultivars, many of them were genetically close to TNS9. TNS9 was developed in 1966 by pure line selection using the introduced line “Giay” from Vietnam, and this variety dominated more than 80% of peanut production in Taiwan in the 1980s because of its favorable flavor after roasting. This variety has also been widely exploited in peanut breeding programs in Taiwan, such as the development of TNG10, HL1, HL2, TN14 and TN18, all produced by the hybridization breeding method. Specifically, TNS9 was directly chosen as a parent of HL1, and indirectly contributed to the genetic background of the other four varieties by being selected as the parent of advanced breeding lines used in the breeding programs of these three varieties. Based on pedigree information, it is thus anticipated that HL2, a Valencia type variety, was grouped into the cluster with mostly Spanish type germplasm. On the other hand, TN16 and TN17, both rich in cyanidine-based anthocyanins on the seed coat, are 2 Taiwanese Valencia type cultivars derived from the same biparental breeding population using the hybridization of 2 landraces collected from central Taiwan. The dendrogram result showed that TN16 and TN17 were clustered into Group III; moreover, they were obviously separated from the other accessions in this clade. Therefore, these two varieties were not closely related to the three groups containing the other 29 accessions, suggesting that potentially locally collected genetic resources can still diversify the current Taiwanese germplasm. The same result of clustering was also found using PCA based on genetic distance (Fig. 2).

### The KASP marker sets identify the population structure better than phenotypes

To understand the genetic diversity beyond the 31 accessions, 14 KASP markers developed by RAD-seq data of 31 accessions were utilized to assess the population structure of 282 peanut accessions from the germplasm conservation center in TARI. On the other hand, we also considered phenotypic data as an alternative tool for the assessment of population structure; specifically, 8 agronomic quantitative traits were evaluated in the field trial in the fall of 2016 using 306 accessions including 282 peanut accessions for the KASP marker validation and 24 out of 31 accessions used in RAD-seq.

The phenotyping results were consistent with similar field trials conducted in India and Turkey, which separated the subsp. *fastigiata* and subsp. *hypogaea* into two groups^46,47^. Similar to the results of two previous studies, we found that the subsp. *fastigiata* accessions in Taiwan had early maturity characteristics. However, our work showed that the subsp. *fastigiata* accessions have more pods than previously reported, their yield-related characteristics indicated the subsp. *fastigiata* accessions produce higher yields than subsp. *hypogaea* accessions in Taiwan (Supplementary Table S4). This result can be explained by the climate in Taiwan which influences the peanut breeding strategy. In terms of climate zones, Taiwan is separated into the north part belonging to the sub-tropical climate zone and the south part belonging to tropical climate zone allowing farmers to annually have two cropping seasons. However, the “plum rain” season between mid-May to mid-June and typhoons occurring between June and October can seriously damage the peanut yield in the end of the first cropping season or the beginning of the second cropping season, respectively. Thus, Taiwanese peanut breeders have chosen peanut accessions with early maturity characteristics, especially Spanish type peanuts, as breeding materials, reflecting the result in Supplementary Table S4 that Spanish type accessions have more pods than accessions from three other market types. For the PCA analyses based on phenotypic data, the 24 accessions used in RAD-seq could not be distinguished, and the 282 accessions used for KASP validation were grouped into two clusters mainly according to the subsp. *fastigiata* and subsp. *hypogaea* (Fig. 4, Supplementary Fig. S1). In the PCA of the 282 accessions, the traits playing the most important roles in PC1 and PC2 were days to flowering, leaf spot resistance level, yield and the number of pods, consistent with traits having significant difference between two peanut subspecies (Supplementary Tables S4, S10).

While we validated these KASP markers using 282 genotypes, the PCA results showed that these genotypes were distinctly separated into 3 groups according to three botanical varieties (Fig. 4). This conclusion was also supported by K-means clustering, in particular with the choice K = 3; beyond that value the BIC value didn’t improve much (Supplementary Fig. S2). In addition, these KASP markers clearly distinguished subsp. *fastigiata* and subsp. *hypogaea* accessions at K = 2, and then separated var. *fastigiata* and var. *vulgaris* from the same subspecies *fastigiata* at K = 3, which was compatible with previous work^22^. However, when setting K = 4, the additional group had a mixture of four market types of germplasm belonging to all three botanical varieties, suggesting that exchanges of genetic background among these accessions may have occurred. This result of a fourth cluster not corresponding to subspecies or market types was also reported in other works^12,42^, and it may result from phenotyping difficulties^48,49^. In both sets, containing respectively 31 and 282 accessions, PCA was used to compare the effectiveness of molecular markers and phenotypic data to cluster samples, and it was demonstrated that PCA based on molecular markers provides more reproducible and satisfactory results than PCA based on phenotypic data (Figs. 2, 4, Supplementary Fig. S1).

## Conclusion

Overall, the genetic diversity and relationship among peanut germplasm in Taiwan was revealed by SNPs identified through the RAD-approach. Our analyses suggest that one should broaden genetic diversity by introducing novel germplasm to prevent genetic vulnerability. In addition, the KASP markers successfully developed here could be useful tools for identifying the population structure of other peanut germplasm collections or for conducting further genetic studies related to breeding.

## Supplementary Information

Supplementary Figures.

Supplementary Tables.

## Data Availability

The sequencing data of 31 accessions produced in this study have been deposited at the NCBI BioProject PRJNA811600. All the codes related to this project are available in the github site https://github.com/ymhsu/ahdivertwn.
