# Supplementary material for: Assessment of genetic diversity and SNP marker development within peanut germplasm in Taiwan by RAD-seq
Source: Sci Rep. 2022 Aug 25;12:14495. doi: 10.1038/s41598-022-18737-0 (PMC9411510; doi:10.1038/s41598-022-18737-0)
Supplement: Supplementary file 1 — Supplementary Figures. [file 41598_2022_18737_MOESM1_ESM.pdf]

Assessment of genetic diversity and SNP marker development within peanut germplasm in Taiwan by RAD-seq

Yu-Ming Hsu[123], Sheng-Shan Wang[4], Yu-Chien Tseng[5], Shin-Ruei Lee[3], Hsiang Fang[3], Wei-Chia Hung[3], Hsin-I Kuo[5], Hung-Yu Dai[3*]

[1]Université Paris-Saclay, CNRS, INRAE, Univ Evry, Institute of Plant Sciences Paris-Saclay (IPS2), 91405, Orsay, France

[2]Université Paris Cité, CNRS, INRAE, Institute of Plant Sciences Paris-Saclay (IPS2), 91405, Orsay, France

[3]Crop Science Division, Taiwan Agricultural Research Institute, 413008, Taichung, Taiwan

[4]Crop Improvement Division, Tainan District Agricultural Research and Extension Station, 71246, Tainan, Taiwan

[5]Agronomy Department, National Chiayi University, 60004, Chiayi, Taiwan

[*]Correspondence:
Hung-Yu Dai
hydai@tari.gov.tw

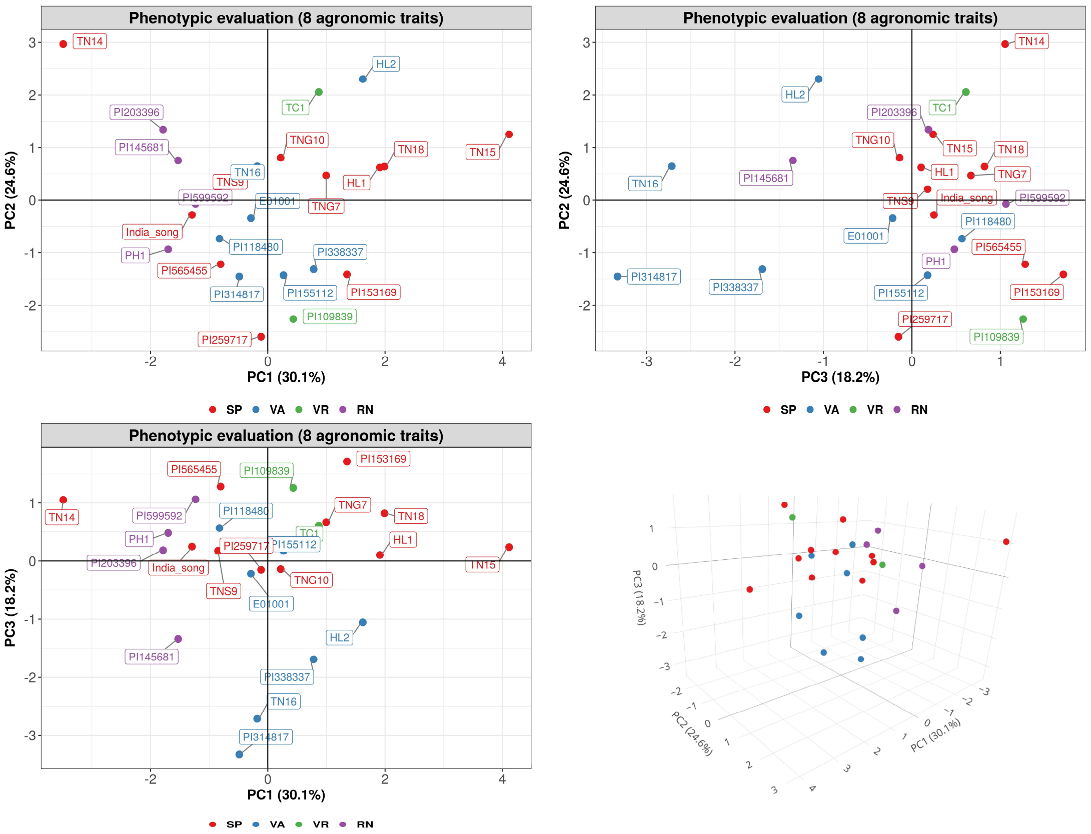

Supplementary Figure 1. Principal component analysis (PCA) of 24 accessions based on phenotypic data. 24 accessions were visualized by 4 market types, Spanish (SP), Valencia (VA), Virginia (VR), Runner (RN).

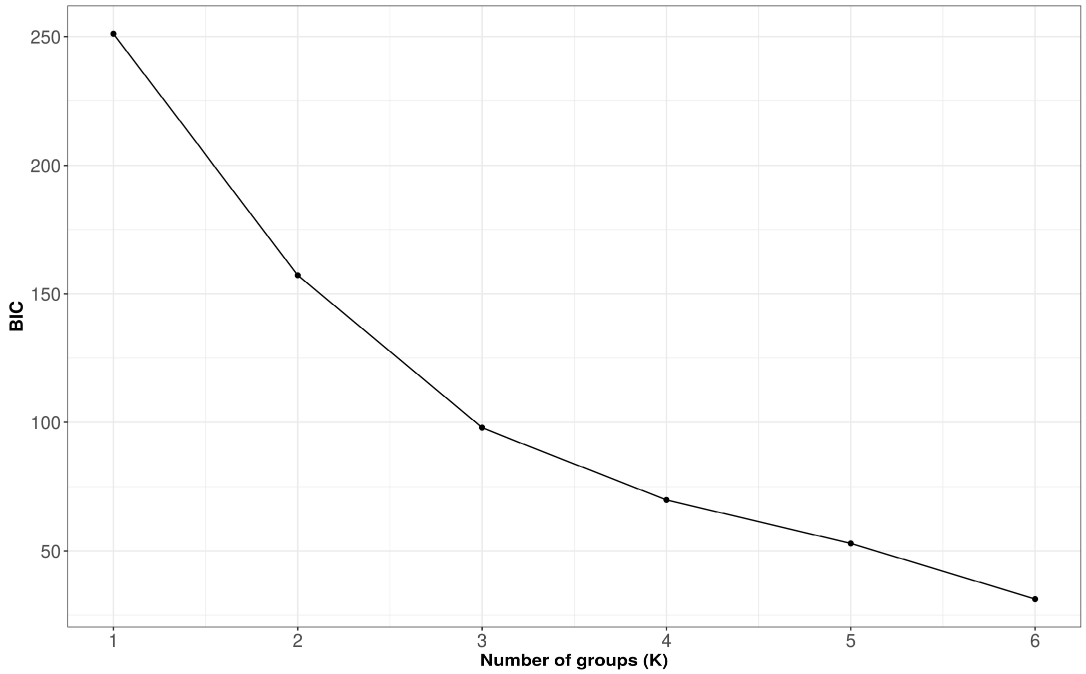

Supplementary Figure 2. The Bayesian information criterion (BIC) value with increasing number of groups (K) based on 282 accessions genotyped by 14 kompetitive allele-specific PCR (KASP) markers.

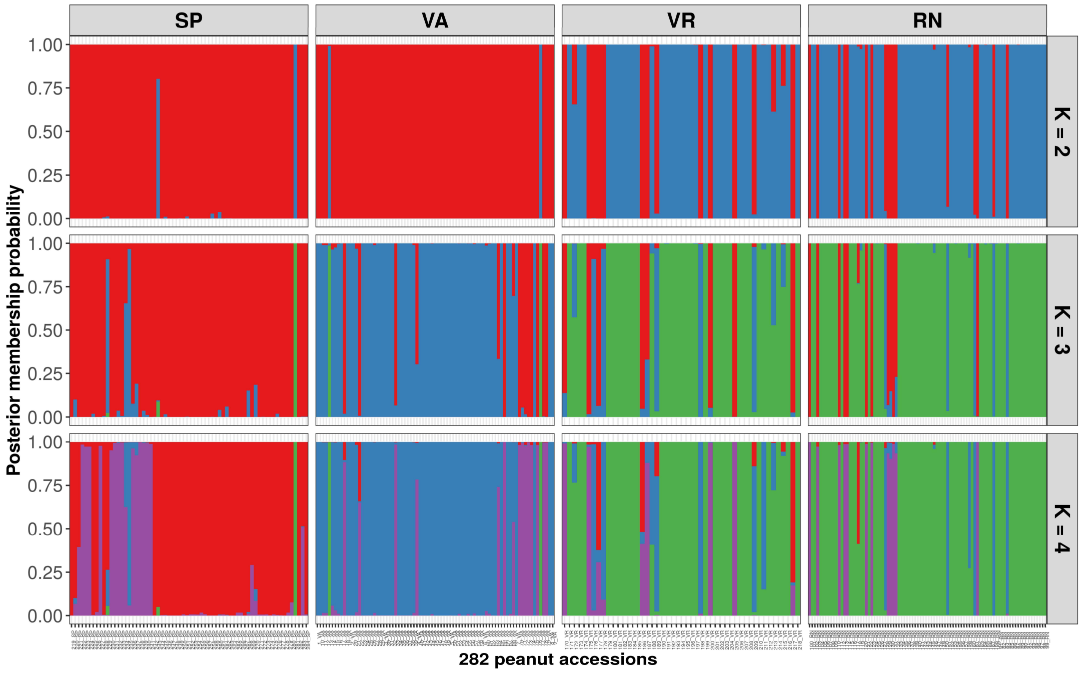

Supplementary Figure 3. Bar plots of posterior membership probability produced by discriminant analysis of principal components (DAPC) for a number of clusters ranging from 2 to 4. The analysis was performed using 282 peanut accessions genotyped by 14 kompetitive allele-specific PCR (KASP) markers, visualized under the classification into the 4 market types: Spanish (SP), Valencia (VA), Virginia (VR), Runner (RN).
